# Optimized Protocol to Generate Spinal Motor Neuron Cells from Induced Pluripotent Stem Cells from Charcot Marie Tooth Patients

**DOI:** 10.3390/brainsci10070407

**Published:** 2020-06-27

**Authors:** Pierre-Antoine Faye, Nicolas Vedrenne, Federica Miressi, Marion Rassat, Sergii Romanenko, Laurence Richard, Sylvie Bourthoumieu, Benoît Funalot, Franck Sturtz, Frederic Favreau, Anne-Sophie Lia

**Affiliations:** 1 CHU de Limoges, Service de Biochimie et Génétique Moléculaire, F-87000 Limoges, France; franck.sturtz@unilim.fr (F.S.); frederic.favreau@unilim.fr (F.F.); anne-sophie.lia@unilim.fr (A.-S.L.); 2 Université de Limoges, Maintenance Myélinique et Neuropathies Périphériques, EA6309, F-87000 Limoges, France; federica.miressi@unilim.fr (F.M.); marion.rassat@gmail.com (M.R.); laurence.richard@unilim.fr (L.R.); sylvie.bourthoumieu@unilim.fr (S.B.); 3 Université de Nantes, RMeS Regenerative Medicine and Skeleton, ONIRIS, INSERM UMR 1229, F-44042 Nantes, France; Nicolas.Vedrenne@univ-nantes.fr; 4 Bogomoletz Institute of Physiology, Department of Sensory Signaling, 01601 Kyiv, Ukraine; S.Romanenko@nas.gov.ua; 5 CHU de Limoges, Service de Neurologie, F-87000 Limoges, France; 6 CHU de Limoges, Service de Cytogénétique, F-87000 Limoges, France; 7 CHU Henri-Mondor, Département de Génétique, F-94000 Créteil, France; benoit.funalot@aphp.fr; 8 Université Paris-Est-Créteil, Inserm U955-E10, F-94000 Créteil, France; 9 CHU de Limoges, UF de Bioinformatique, F-87000 Limoges, France

**Keywords:** induced pluripotent stem cells, hiPSC, spinal motor neurons, cellular models, peripheral nervous system, Charcot-Marie-Tooth, CMT, peripheral neuropathy

## Abstract

Modelling rare neurogenetic diseases to develop new therapeutic strategies is highly challenging. The use of human-induced pluripotent stem cells (hiPSCs) is a powerful approach to obtain specialized cells from patients. For hereditary peripheral neuropathies, such as Charcot–Marie–Tooth disease (CMT) Type II, spinal motor neurons (MNs) are impaired but are very difficult to study. Although several protocols are available to differentiate hiPSCs into neurons, their efficiency is still poor for CMT patients. Thus, our goal was to develop a robust, easy, and reproducible protocol to obtain MNs from CMT patient hiPSCs. The presented protocol generates MNs within 20 days, with a success rate of 80%, using specifically chosen molecules, such as Sonic Hedgehog or retinoic acid. The timing and concentrations of the factors used to induce differentiation are crucial and are given hereby. We then assessed the MNs by optic microscopy, immunocytochemistry (Islet1/2, HB9, Tuj1, and PGP9.5), and electrophysiological recordings. This method of generating MNs from CMT patients in vitro shows promise for the further development of assays to understand the pathological mechanisms of CMT and for drug screening.

## 1. Introduction

Peripheral nerves are critical for the functioning of the nervous system, as they forward information to the spinal cord and encephalon and provide the periphery (muscles, organs, skin, blood vessels) with adapted signals. As such, peripheral nerve illnesses constitute an important source of medical problems and a large group of neurological diseases of various origins. Among them, hereditary peripheral neuropathies, such as Charcot–Marie–Tooth disease (CMT) disease, have a prevalence of 1:2500 and often affect patients during their entire lives. The use of next-generation sequencing has tremendously improved the molecular diagnosis of these diseases in recent years by efficiently determining the mutations involved. However, even when a gene mutation is identified, the molecular and cellular pathways involved in the pathophysiology remain difficult to decipher. Because of the numerous mutations and various genes involved, animal models are of limited utility and are highly difficult to study, aside from the potential ethical problems. As an alternative, in vitro cellular models appear to be a promising path for the expeditious development of therapeutic strategies. 

In this context, human-induced pluripotent stem cells (hiPSCs) generated from patients, associated with their ability to differentiate toward the cell type of interest, appear to be a potentially powerful tool, as it is very difficult, if not impossible, to study live peripheral nerve cells. The work of Yamanaka et al. on iPSCs opened the way to creating dedifferentiated cells and, later, to observing the behavior of previously unattainable cells [1,2]. They showed that the reprogrammation of dermal fibroblasts using non-integrative plasmids that included the Oct4, Sox2, Klf4, and l-Myc genes induces hiPSCs that can be subsequently differentiated into many cells types [3]. The differentiation of hiPSCs into neuronal cells is an essential step.

Several protocols have been developed to differentiate human embryonic stem cells (hESCs) [4,5] or hiPSCs [6,7,8,9,10] into spinal motor neurons (MNs). In the peripheral neuropathy field and, in particular, in CMT diseases, several groups have attempted to obtained spinal MNs [11] and more recently differentiate hiPSCs into Schwann cells [12,13]. However, cells from patients are not always easily reprogrammed and differentiated. Based on an extensive review of the literature and results obtained in our laboratory, we developed a robust and reproducible protocol to improve MNs differentiation of hiPSCs obtained from CMT patients.

## 2. Materials and Methods

### 2.1. Cell Culture Media

iPSC medium: KO-DMEM (Life Technologies, Carlsbad, CA, USA), supplemented with 20% KnockOut Serum Replacement (Life Technologies), 1X MEM non-essential amino acids (Life Technologies), 2 mM Glutamine (Life Technologies), 50 µM β-mercaptoethanol (Life Technologies), and 10 UI/mL gentamycin (Life Technologies).

Differentiated medium: DMEM/F12 (Life Technologies), 2% B27 without vitamin A (Life Technologies), 5 µg/mL heparin (Sigma-Aldrich, Saint-Quentin Fallavier, France), and 100 µM β-mercaptoethanol (Life Technologies).

Neural induction medium: 1:1 DMEM/F12 (Life Technologies) and Neurobasal A (Life Technologies), 1% N2 supplement (Life Technologies), 2% B27 without vitamin A (Life Technologies), and 100 µM β-mercaptoethanol (Life Technologies).

### 2.2. Experimental Design

#### 2.2.1. Generation of hiPSCs

HiPSCs were obtained as previously described [14]. Briefly, human dermal fibroblasts, negative for HVB, HVC, and HIV virus (Hospital virology department, Limoges, France) and mycoplasma (MycoAlert mycoplasma detection kit, Lonza) were used to generate hiPSCs. Three plasmids (Plasmid #6: pCXLE-hOCT3/4 shp53-F Addgene (Watertown, Massachusetts, USA), Plasmid #7: pCXLE-hSK Addgene, and Plasmid #8: pCXLE-hUL Addgene) at 1 µg/mL were used to reprogram fibroblasts into hiPSCs using a Nucleofector II device (Amaxa, Lonza AAD-10015, (Bâle, Switzerland). Directly after nucleofection, 100,000 cells were seeded on mitomycin mouse embryonic fibroblasts with culture medium consisiting of DMEM GlutaMAX (Life Technologies) supplemented with 10% fetal bovine serum (FBS) (Life Technologies) and 1X MEM non-essential amino acids (Life Technologies) and incubated at 37 °C in a water-saturated atmosphere with 5% CO_2_. At day 1, the medium was replaced by the same culture medium supplemented with 10 UI/mL gentamycin (Life Technologies). At day 4, the culture medium was replaced with hiPSC medium and this medium changed every day up to day 10. Then, hiPSC colonies were picked 2–4 weeks post-nucleofection. Fifteen days after nucleofection, the morphology of the fibroblasts changed to form colonies with a typical morphology (Figure S1). Approximately 40 colonies per patient or control were isolated for further expansion. HiPSC colonies were cultivated on mitomycin mouse embryonic fibroblasts (CF 1 MEF 4M Mito C, TebuBio) seeded on 0.1% gelatin (G1393-100ML, Sigma-Aldrich, Merck). Every day, hiPSC colonies were cleaned to remove the differentiated cells using a needle (26G, Dutscher, Brumath, France) and the culture medium was changed with complete fresh hiPSC medium supplemented with 20 ng/mL FGF2 (fibroblast growth factors). The hiPSCs were characterized at passage 15 (Figure S2, Appendix A). 

The CMT2 patients included in this study were two men of three and 23 years of age. The younger patient carried a homozygous nonsense mutation in *GDAP1* (p.Ser194*, c. 581C>G). He developed a severe form of CMT2 with multiorgan failure, leading to an early death at three years of age. The older patient has a different homozygous nonsense mutation in *GDAP1* (p.Gln163*, c. 487C>T) and is currently using a wheelchair. The first signs of the disease appeared during his childhood, with motor problems observed more in the lower than upper limbs, followed by sensitive troubles. No response was obtained when muscles were stimulated during an electromyogram. The healthy controls consisted of three women and two men (ranging from 24 to 56 years of age) without peripheral neuropathy nor any mutation in *GDAP1*, investigated by sequencing (data not shown).

#### 2.2.2. Generation of Motor Neurons

**Figure 1:**
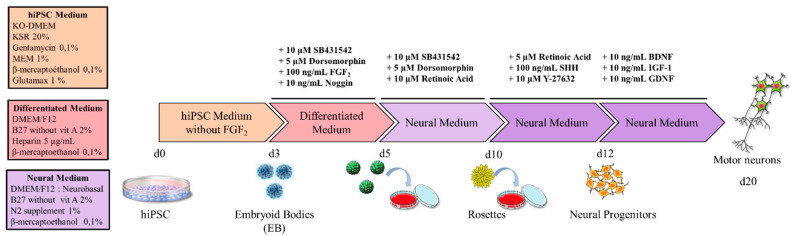
Schematic representation of motor neuron induction with all media and factors.

**Figure 2:**
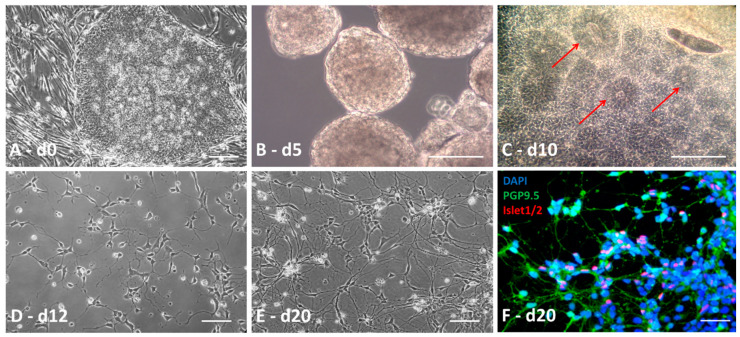
Induction of spinal motor neurons. HiPSc colonies (**A**) were cut into large squares to generate EBs (embryoid bodies) (**B**) and grown in classical medium for three days. EBs evolved in differentiated medium for two days and were seeded on a poly-L-ornithin/laminin plate in neural induction medium up to the apparition of rosettes (**C**, red arrows). Rosettes were gently manually removed and dissociated. Single cells were seeded on poly-L-ornithin/laminin dishes at 100,000 cells/cm^2^ to generate neuronal precursors (**D**). Neuronal precursors were then dissociated and seeded at 20,000 to 40,000 cells/cm^2^ (**E**) to generate motor neurons. The proportion of motor neurons increased from 10% to 80% following maturation from day 15 to day 20 ((**F**) 4′,6′-diamidino-2-phénylindole dihydrochloride (DAPI) in blue, PGP9.5 in green, and Islet cocktail in red). Scale bar = 50 µm.

The protocol is summarized in the scheme in Figure 1. At day 0, hiPSC colonies were cut into homogenous squares using a StemPro^®^ EZPassage™ (Life Technologies; Figure 2A). Colonies were collected and suspended in 60-mm ultralow-attachment dishes (Corning Incorporated, New York, NY, USA) in 5 mL hiPSC medium without FGF2. At day 1, the medium was changed by sedimentation: dead cells were discarded with the supernatant, whereas sedimented cells were transferred to a new 60-mm ultralow-attachment dish using 5 mL fresh iPSC medium. At day 3, when embryoid bodies (EBs) are fully formed, differentiation medium was applied, extemporaneously supplemented with 10 µM SB431542 (Tocris Bioscience, Minneapolis, MN, USA), 5 µM Dorsomorphin (Sigma-Aldrich, Merck), 100 ng/mL FGF2 (PeproTech Inc., Rocky Hill, NJ, USA), and 10 ng/mL Noggin (PeproTech Inc.). The culture medium was renewed daily up to day 5 and detached cells in the supernatant were isolated by sedimentation and plated in a new dish as already described (Figure 2B). From day 5, the cells required a specific coated-plate. Thus, the plate was incubated with 20 µg/mL poly-L-ornithine (Sigma-Aldrich, Merck) for 4 h at 37 °C. Excess poly-L-ornithine in the dish was discarded and the plate dried for 30 min at room temperature. After washing three times with sterile water or saline buffer, the dish was dried at room temperature (opened under the hood). For the upper coating, laminin (Invitrogen, Thermo Fisher Scientific) was diluted with fresh neural induction medium (without supplement) to a final concentration of 20 µg/mL. The solution was added to cover the entire dish surface and incubated overnight at 37 °C. On days 5–7, EBs were sequentially seeded on the 60-mm coated dishes in 5 mL neural induction medium supplemented with 10 µM SB431542 (Tocris Bioscience), 5 µM Dorsomorphin (Sigma-Aldrich), and 10 µM retinoic acid (RA) (Sigma-Aldrich). Prior to treatment, EBs needed to be of the same size, circular, smooth, and brownish, without black spots, to maximize the efficiency of the protocol. Every two days, fresh supplemented neural induction medium was added to the dish until “rosette” formation. Mature “rosettes” were observed on day 10 (Figure 2C). 

“Rosettes” were isolated from the other cells using a simple needle to make the smallest squares possible and collected in a tube containing a small volume of Dulbecco’s phosphate-buffered saline (DPBS) to wash them. Trypsin solution (Gibco, Thermo Fisher) was added to facilitate cell dissociation. After 5 min of incubation at 37 °C in a water-saturated atmosphere and 5% CO_2_, “rosettes” were gently mechanically dissociated under the microscope until a homogeneous cell suspension was obtained. Then, fresh neural induction medium containing 10% FBS was added to the suspension to stop enzyme activity. After centrifugation at 200× *g* for 5 min, the supernatant was discarded and the cells plated at 100,000 cells per cm^2^ in a 96- or 48-well plate coated with 20 µg/mL poly-L-ornithine and 3 µg/mL laminin (Figure 2D). The neural induction medium was supplemented with 100  ng/mL Sonic Hedgehog (Shh) (PeproTech Inc.), 5 µM RA, 10 µM Y-27632 ROCK inhibitor (Calbiochem, Billerica, MA, USA), 10 ng/mL BDNF (brain-derived neurotrophic factor), 10 ng/mL GDNF (glial cell line-derived neurotrophic factor), and 10 ng/mL IGF-1 (insulin-like growth factor-1) (PeproTech Inc.) to generate neuronal precursors. This supplemented culture medium was renewed every two days, except for the Y-27632 ROCK inhibitor, which was added only after passing the cells. The neural progenitors needed to be passed every 3–4 days using the trypsin method, as already described. Neuronal precursors were plated at a density of 20,000 to 40,000 cells/cm^2^ in the same supplemented medium to generate completely differentiated MNs. First, neurites were observed 24 h after plating (Figure 2E,F). Neural precursors may also be stored by freezing at this stage. Briefly, they were collected as described, centrifuged for 5 min at 200× *g* and cryopreserved in CryoStor CS10 (Stemcell Technologies, Grenoble, France) added to the cell pellet. The frozen vials were then stored long-term in standard liquid nitrogen storage containers. 

### 2.3. Immunostaining 

Staining was performed to characterize hiPSCs and effective neuronal differentiation. Cells were fixed in 4% paraformaldehyde (Sigma-Aldrich, Saint-Quentin Fallavier, France) for 10 min at room temperature and rinsed three times with 1X DPBS for 5 min. The cells were permeabilized with 0.2% Triton X-100 (Sigma-Aldrich, Saint-Quentin Fallavier, France) and 3% bovine serum albumin (BSA) in 1X DPBS for 1 h at room temperature. Cells were washed and incubated with primary antibodies in 3% BSA overnight at 4 °C (Table 1). Cells were subsequently labeled with the appropriate fluorescently-tagged secondary antibodies, Alexa fluor 488 (green fluorescence) and Alexa fluor 594 (red fluorescence) (Molecular Probes, Eugene, OR, USA). Cells were then counterstained with 1 mg/mL 4′,6′-diamidino-2-phénylindole dihydrochloride (DAPI, Sigma-Aldrich) to stain the nuclei. Cells were observed with a fluorescence microscope (Leica DM IRB, Nanterre, France) and a confocal microscope LSM 880 (Zeiss, Germany). Images were obtained using NIS Element BR and Zen software and treated with image J software (NIH, Bethesda, MD, USA).

### 2.4. Electrophysiology

Cells were covered with an approximately 1.5-mm-thick fluid layer (Saline solution, Live Cell Imaging Solution, Life Technologies) and placed under an inverted microscope (IX70, Olympus, Shinjuku, Tokyo, Japan). Cells were illuminated with an upright microscope condenser and a 4x objective was used to distinguish the neuronal shapes. For electrophysiological recordings, patch electrodes were generated by pulling borosilicate capillary glass (1.5/0.75 mm OD/ID, 1B150F-4, WPI, Sarasota, FL, USA) associated with a microelectrode filled with an intracellular solution with a resistance between 3 MOhm and 4 MOhm. The solution composition was: 140 mM K-gluconate, 10 mM HEPES, 2 mM Mg-ATP, and 1.1 EGTA, with the pH adjusted to 7.3 with KOH; the sodium channel recording solution composition was: 135 mM Cs-gluconate, 5 mM CsF, 10 mM HEPES, 2 mM Mg-ATP, and 1.1 EGTA, with pH adjusted to 7.3 with CsOH. All electrophysiological recordings were performed using a microelectrode amplifier (PC-ONE Patch/Whole Cell Clamp, CORNERSTONE Series, Dagan, USA) in the voltage-clamp mode, with a holding potential of −70 mV in the whole-cell configuration. Acquired transmembrane current alterations were digitized online at 20 kHz after passing through a low-pass Bessel filter with the setting at 10 kHz using data acquisition hardware (DigiData 1440A; Molecular Devices) and software (Whole Cell Electrophysiology Analysis Program V4.8.2, (c) John Dempster, University of Strathclyde 1996–2014). Leak current and stray capacitance were instrumentally pre-compensated and residual capacitance and related artifacts were subtracted using the P/N method. Electrophysiological recordings were performed using the Stimulus Protocol mode and processed offline using data analysis software (Whole Cell Electrophysiology Analysis Program V4.8.2, (c) John Dempster, University of Strathclyde 1996–2014, and OriginPro 8). Whole-cell currents were measured in response to the voltage ramp command protocol from −80 mV to 50 mV (with a rate of voltage augmentation of 0.65 mV/msec) (*n* = 4).

## 3. Results

### 3.1. Obtaining iPSCs from Patients

We launched this study to define a robust protocol to obtain and differentiate cells obtained from CMT patients into MNs. The hiPSCs from five healthy controls and two CMT2 patients were generated and characterized according to a procedure developed by iStem (INSERM/UEVE UMR 861, AFM, Genopole, Evry, France) (Figures S1 and S2 and Appendix A). At this step, there were no observable morphological differences between hiPSCs from the healthy controls and patients (data not shown). However, it was more difficult to obtain the hiPSCs from the CMT2 patients than the controls, perhaps due to the mutation (one patient carrying a *GDAP1* homozygous nonsense mutation p.Gln163*, c. 487C > T and the other a *GDAP1* homozygous nonsense mutation p.Ser194*, c. 581C > G). Nevertheless, it was possible to obtain hiPSCs from both.

### 3.2. Definition of the Factors and the Timeframes for MN Differentiation

**Figure 3:**
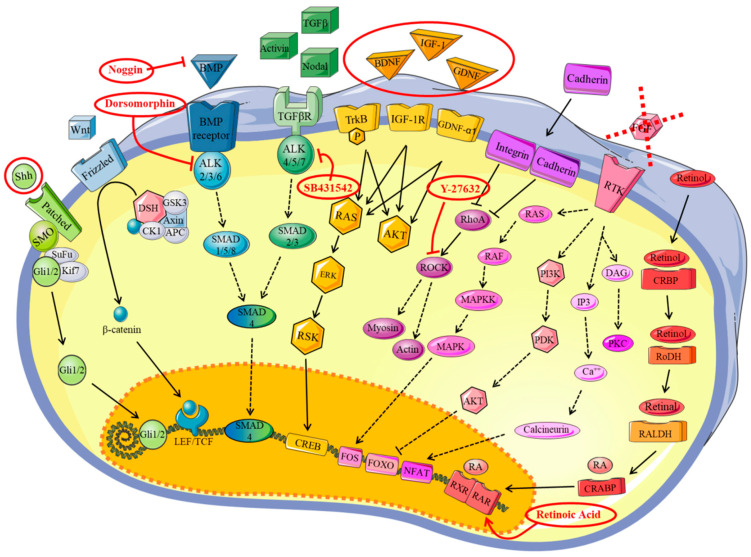
A schematic representation of the trophic factor pathways used for motor neuron differentiation in this study. Sonic Hedgehog (Shh) pathway in green [17]; Wnt pathway in clear blue [18]; BMP (morphogens bone morphogenetic proteins) pathway in dark blue [19]; transforming growth factor-*β* (TGF-*β*) pathway [20]; BDNF (brain-derived neurotrophic factor), IGF-1 (insulin-like growth factor-1), and GDNF (glial cell line-derived neurotrophic factor) pathways in yellow [21,22,23]; Rock inhibitor pathway in purple [24]; FGF2 (fibroblast growth factors) pathway in pink [25]; retinoic acid pathway in red [26]. Red circles indicate the trophic factors used in this study.

We first tested various published protocols to generate MNs from our iPSCs but with limited success. This led us to test several conditions and factors. We first investigated the factors involved in embryonic development towards the neuronal lineage. According to the literature [7,11,15,16], Shh and RA are key factors. However, the MN differentiation rate was still too low (20%) when we tested these factors. To increase the MN differentiation rate, we tried other factors, in addition to RA and Shh, such as Noggin, dorsomorphine, BDNF, IGF-1, GDNF, SB431542, and Y-27632, with the aim to activate different pathways involved in differentiation, as described in Figure 3. After numerous attempts (up to six months), we defined an optimized protocol that enables the generation of MNs in 20 days with a MN differentiation rate of approximately 80%. The factors, concentrations, and timepoints are given in the Materials and Methods section and summarized in Figure 1.

### 3.3. Differentiation into Motor Neurons

**Figure 4:**
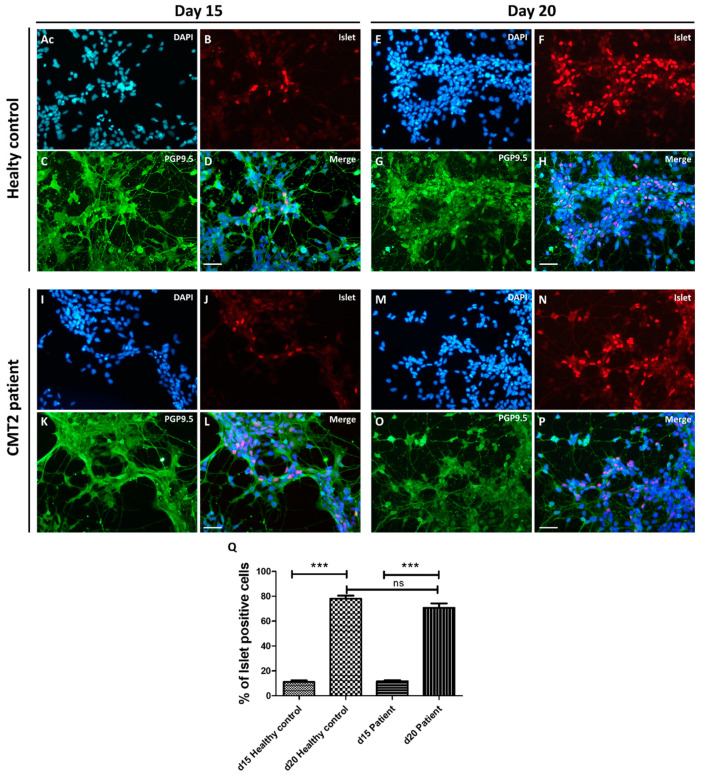
hiPSC differentiation into motor neurons at d15 and d20 from healthy control (**A**–**H**) and CMT2 patient (**I**–**P**). Immunocytochemistry was performed at d15 and d20. Nuclei were stained with DAPI (blue **A**,**E**,**I**,**M**), neurons with PGP9.5 (green **C**,**G**,**K**,**O**), and motor neurons with an Islet cocktail (red **B**,**F**,**J**,**N**). At day 15, all cells were differentiated into neurons (100% PGP9.5) and 10% were Islet positive (**A**–**D**,**I**–**L**). Five days later (day 20), the proportion of motor neurons increased to up to 80% due to a maturation process (**E**–**H**,**M**–**P**). Scale bar = 50 µm. Histograms showed the progressive maturation of cells to the motor neuron (MN) phenotype between d15 and d20 for healthy control and patient (**Q**) (Student T Test, *n* = 4 to 7, *** *p* < 0.001).

**Figure 5:**
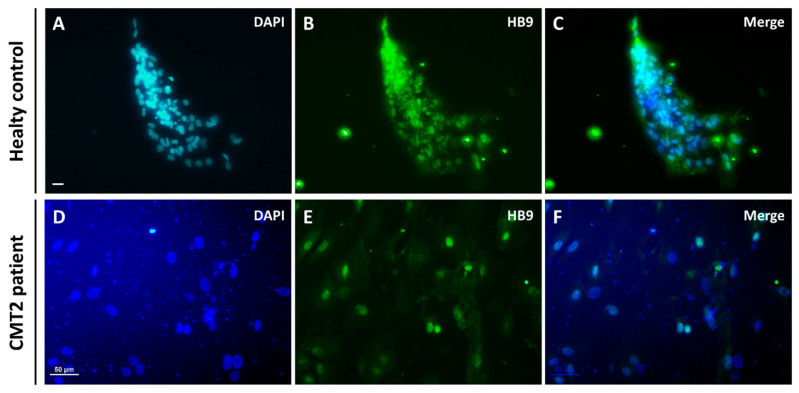
hiPSC differentiation into motor neurons at d20. Nuclei were stained with DAPI (blue **A**,**D**) and motor neurons with HB9 (green **B**,**E**). **A**–**C** show immunocytochemistry for healthy control cells and **D**–**F** for CMT2 patient cells. Scale bar = 50 µm.

MNs were generated by dissociating and seeding neuronal precursors at 20,000 cells/cm^2^ to 40,000 cells/cm^2^ in supplemented differentiation medium (Figure 2E). After five days, spinal cells were characterized by immunochemistry (Figure 4A–D,I–L). All cells expressed PGP9.5 and 10% were Islet positive, which are specific markers of neuronal cells and MNs, respectively. Five days later, the proportion of MNs increased to up to 80% due to a maturation process (Figure 4E–H,M–Q)) and 80% of the cells were HB9 positive, which is a specific nuclear label of MNs, thus confirming their ventral spinal cord phenotype (Figure 5A–F). The expression of Islet at d20 was not significantly different between the control and patient groups (Student t test). Moreover, ChAT immunostaining was performed at d15 on neuronal progenitors from a healthy control. The neuronal progenitors already expressed ChAT (approximately 31%), suggesting that our MNs may be cholinergic (Figure S3). Based on the immunocytochemistry results (Figure 4D,H,L,P) and visual observation by optic microscopy (Figure 2E,F), the morphology appeared to be typical of MNs, with long processes, a small soma, and a process network. Thus, Ki-67 immunostaining was performed on the neuronal progenitors from a healthy control. Only 32% of the cells were Ki-67 positive versus 87% at the iPSC stage, in support of a weak ratio of progenitors undergoing cell proliferation (Figure S4).

**Figure 6:**
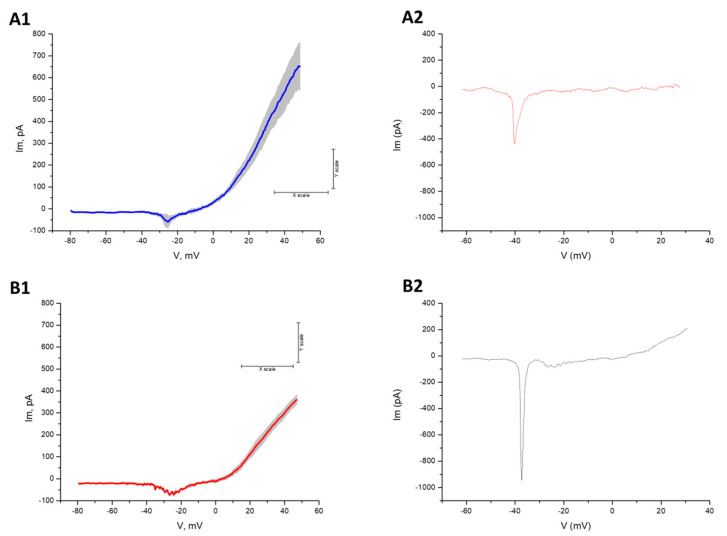
Healthy control (**A1** and **A2**) and CMT2 patient (**B1**–**B2**) motor neuron electrophysiology (*n* = 4). (**A1**,**B1**) Averaged traces with SE shadow, showing a persistent inward current and strong outward current. (**A2**–**B2**) Individual traces obtained with high a Cs^+^ inside solution, showing a fast transient inward current.

We performed electrophysiological recordings to estimate alterations in transmembrane currents in both control and patient MNs (Figure 6). The typical electrophysiological characteristics of MNs were compared between groups and are supported by previous studies [11,27]. In particular, CMT2-patient derived MNs showed stronger inward currents, in the range of 40–30 mV, and weaker outward currents than the control group. Both differences are consistent with previously reported intrinsic hyperexcitability for CMT MNs [11].

## 4. Discussion

We aimed to create a robust protocol to obtain hiPSCs differentiated into MNs from CMT patients. We also aimed to use only a limited number of factors that mimic embryonic development to stimulate the various pathways (summarized in Figure 3) within a defined timeframe. This protocol allowed us to obtain 100% cells expressing neuronal markers and 80% spinal MNs in only 20 days. MNs could be used up to d30 without any sign of degeneration.

We returned to embryology to handpick efficient differentiating factors. Indeed, the balance between activation and inhibitory pathways during embryonic development must be understood to choose MN-inducing factors (Figure 3) [28,29,30]. Neural tube development follows two axes (Figure S5A1). Dorso-ventral differentiation is controlled by opposite gradients of the morphogens bone morphogenetic proteins (BMP) and Shh. Approximately 30 types of BMPs (family members of transforming growth factor-*β* (TGF-*β*)) are expressed during embryonic development [31,32] and their activities are inhibited by three molecules from the chord: noggin, chordin, and follistatin, promoting neural-tube formation [31,33]. Shh is synthesized by the notochord and neural-tube floor, promoting interneurons and MN differentiation (Figure S5A1,A2). Specific transcription factors, such as Pax6, Olig2, Nkx6.2, and Nkx6.1 are stimulated by these gradients [34,35]. Antero-posterior differentiation is based on morphogen signals through RA, FGF, or Wnt production by the axial and paraxial mesoderma and endoderma [36]. The gradient is distributed from the caudal to cranial section and is involved in modification of the hindbrain or, in the anterior section, the spinal cord [35,37] (Figure S5B). We applied this knowledge to define the best factors.

Few protocols have been proposed for MNs differentiation, all showing various rates. Wichterle et al. reported the differentiation of mouse ESCs into MNs using RA and Shh agonists [15], with a rate of differentiation of 20% to 40%. Their protocol was modified by Miles et al. and the proportion increased to 60% to 80% using N2 supplement in the culture medium [16]. During differentiation, human rosettes appear later than those of mice and a specific cocktail containing BDNF [21,38], IGF-1 [22,39,40,41], and GDNF [23,42] was found to be essential for the survival and growth of neural progenitors. However, the proportion of MNs obtained by Singh Roy et al. was approximatly only 10% after 28 days and 50% after 35 days [5]. Dimos et al. generated MNs using Shh and RA for amyotrophic lateral sclerosis [7]. They reported that 20% of the cells expressed HB9, a cholinergic neuronal marker, of which more than 90% expressed Islet1/2 [7]. Based on a study of Watanabe et al., we chose to add a ROCK inhibitor (Y-27632) to protect cells from apoptosis and promote neuronal differentiation [24]. Hu et al. obtained MNs in 35 days and functionally mature MNs were generated in 56–70 days, with a final proportion of approximately 50%. Interestingly, at the MN generation step, the cells required limited concentrations of Shh and RA to prevent inhibition of MN differentiation [9]. After 14 days, Chambers et al. obtained 30% Islet-positive cells and 60% were HB9 positive [6]. Kim et al. reported that SB431542 and dorsomorphin (used in our protocol), inhibitors of both the activin/nodal and BMP pathways, improved the neural differentiation of hESCs and hiPSCs by more than 90% [10]. This led us to supplement the medium with the following factors: RA, Shh, Noggin, dorsomorphine, BDNF, IGF-1, GDNF, SB431542, and Y-27632.

Only a limited number of groups have worked on MNs derived from hiPSCs in CMT disease, despite the fact that more than 90 genes are involved [43,44]. In the realm of axonal CMT, Saporta et al. and Juneja et al. studied the *NEFL*, *MFN2*, *HSPB8*, and *HSPB1* mutations [11,45]; Ohara et al. studied those of *MFN2* [46]; and Kim et al. studied those of *HSPB1* [47], whereas nobody has studied mutations of *GDAP1*, according to our knowledge. In their protocol, Saporta et al. used SMAD signaling inhibition, Shh, and RA [11], obtaining mature spinal MNs in 35 days, as in the protocol of Ohara et al. [46]. Kim et al. [47] obtained mature spinal MNs in 21 to 28 days. We obtained mature spinal MNs in 20 days using our protocol. The ratio of spinal MNs was not mentioned in these publications and this has presented problems in deciphering the mechanisms involved in this disease and in performing drug screening, as not all the cells were MNs. 

In the future, it may be worth testing the use of a Wnt pathway activator, as demonstrated, to further increase the rate of MN differentiation [18]. It may aso be worth would testing calcitriol, as it has a synergic effect with Wnt, Shh, or Klotho, and binds to the vitamin D receptor, which is associated with the nuclear receptor of RA [48]. In addition, calcitriol is involved in various processes, such as neural stem cell differentiation, axon genesis, and the growth of MNs [48]. Furthermore, the high purity of the cells obtained with this protocol could be further improved by sedimentation field flow fractionation (SdFFF), which makes it possible to obtain neural and endothelial precursors after spontaneous differentiation in basic medium, with no added factors [49].

Finally, we defined the cells we obtained as spinal MNs by morphological observation, immunostaining for seveal markers, and electrophysiological recordings. This characterization is already convincing. However, it would be informative to co-culture these MNs with myotubes or Schwann cells to verify that they function properly.

We applied this efficient protocol to three controls (two women and one man, ranging from 24 to 56 years of age) and two patients (two males, three and 23 years of age) to generate mature MNs, supporting this robust method. We believe that this protocol could also be applied to various types of patient cells (various ages and different sex) and used to obtain hiPSCs from CMT patients with different gene variations (*MFN2*, *PMP22*, etc.).

The optimized method that we have developed to generate MNs provides a true opportunity to discover new therapeutics. With this in vitro model, the screening of potential therapeutic molecules is possible, directing efficient molecules towards animal models and clinical trials. This model is also relevant for generating MNs or neural progenitors harboring various mutations from the fibroblasts of patients for an injection of their corrected cells by the promising CRISPR Cas9 technique. This method could be applied not only to CMT patients but also those with other genetic peripheral neuropathy diseases.

## 5. Conclusions

This protocol should aid researchers to easily and rapidly differentiate hiPSCs into MNs (only 20 days for the first MNs) using a limited number of growth factors, with a high success rate (approximately 80% vs 10% to 60% for other protocols). This technique to derive MNs from hiPSCs is a critical step to mimic neurological diseases of genetic origin, such as CMT, in vitro. These models will allow investigation of the molecular pathways involved in the disease and, hopefully, help in the development of new therapeutic strategies, particularly as a tool for drug screening. 

## Figures and Tables

**Table 1:** Primary antibodies used for human-induced pluripotent stem cell (hiPSC), neuron, and motor neuron characterization.

Antibody	Company	Cat Num	Species/Type	Dilution
Pluripotency				
Nanog	Abcam	130095632	Rabbit poly IgG	1:100
Oct3/4	Santa Cruz Biotech	sc-5279	Mouse Mono IgG2B	1:100
Sox2	Chemicon	AB5603	Rabbit poly IgG	1:100
Spontaneous Differentiation in Three Germinal Layers				
Pax6	Covance	PRB-278P	Rabbit poly IgG	1:100
αSMA	DAKO	M0851	Mouse IgG2A	1:500
Sox17	R&D	AF1924	Goat IgG	1:100
Neuronal and Motor Neuronal				
Tuj1	R&D	MAB1195	Mouse Mono IgG	1:500
PGP9.5	Ultraclone	Ra95101	Rabbit poly IgG	1:500
HB = MNR2	DSHB	81.5C10	Chicken	1:100
Islet1	DSHB	40.2D6-c	Mouse Mono IgG	1:25
Islet1/2	DSHB	39.4D5-c	Mouse Mono IgG	1:25
ChAT	Chemicon	AB144P	Goat IgG	1:20
Other				
Ki-67	Leica	NCL-L-Ki67-MM1	Mouse Mono IgG	1:200

## Supplementary Materials

The following are available online at https://www.mdpi.com/2076-3425/10/7/407/s1, Figure S1. Schematic representation of hiPSC induction. Dermal fibroblasts were reprogrammed with Yamanaka’s cocktail (Oct4, Sox2, Klf4, c-Myc) into induced pluripotent stem cells. HiPSC clones were generated in 18 days and then amplified to passage 15; Figure S2. hiPSC generated from a healthy control (A1–P1) and a CMT2 patient (A2-P2). HiPSC colonies have a typical morphology (A1, A2), with a nucleus/cytoplasma ratio of 1:1. Embryoïd bodies (B1, B2) could be differentiated into cell types from the three embryonic germ layers following spontaneous differentiation (C1, C2) and labelling with α-SMA ((E1, E2) mesoderma), PAX6 ((F1, F2) ectoderma), and Sox17 ((G1,G2) endoderma). HiPSCs expressed pluripotency markers, including Nanog, Oct3/4, and Sox2 (I1-P1, I2, P2), were positive for alkaline phosphatase (D1, D2), and had normal karyotypes (H1, H2); Figure S3. ChAT immunostaining performed at d15 on neuronal progenitors from a healthy control; Figure S4. Ki-67 immunostaining performed at d0 (iPSC stage A-B, E) and d15 (neuronal progenitor C-D, E) from a healthy control. (E) Histograms showed the KI-67 positives cells betwenn hiPSCs (d0) and neuronal progenitor (d15) from a healthy control (Student T Test, *n* = 4 to 7, *** *p* < 0.001); Figure S5. Factors involved in dorsal-ventral polarity during cord differentiation (A), factors involved in antero-posterior differentiation during neurulation (B), and MN markers during differentiation (C) adapted from Casarosa et al., 2013 and Davis-Dusenbery et al., 2014 [35,37].

## Appendix A. HiPSC Characterization

a. Morphology

The HiPSC colonies, EBs, and spontaneous differentiation were observed by light microscopy. The images were captured with a Nikon D90^®^ digital camera.

b. The alkaline phosphatase

Alkaline phosphatase was determined according to the manufacturer’s guidelines (Sigma, Fast BCIP/NBT SIGMA B5655).

c. Karyotyping

Metaphase cells were obtained after cell-cycle arrest in mitosis by the addition of demecolcine solution (10mg/mL, Sigma) to the cultures for 2 h after the end of exposure. Mitotic arrest was followed by treatment with a hypotonic solution (diluted fetal calf serum) to increase the cell volume and disrupt cell membranes. The cells were then fixed in 3:1 methanol:acetic acid before being spread onto microscope slides. Chromosomes were stained by the R-banding method, which produces a pattern of bands on chromosomes. R-banding was obtained by heating slides at 88 °C in Earle’s buffer followed by Giemsa staining. Metaphase cells were observed under the microscope (Zeiss Axioplan 2 imaging) and karyotyped using the CytoVysion image analysis system (Applied Imaging).
