# Supplementary material for: Optimized Protocol to Generate Spinal Motor Neuron Cells from Induced Pluripotent Stem Cells from Charcot Marie Tooth Patients
Source: Brain Sci. 2020 Jun 27;10(7):407. doi: 10.3390/brainsci10070407 (PMC7408498; doi:10.3390/brainsci10070407)
Supplement: Supplementary file 1 [file brainsci-10-00407-s001.pdf]

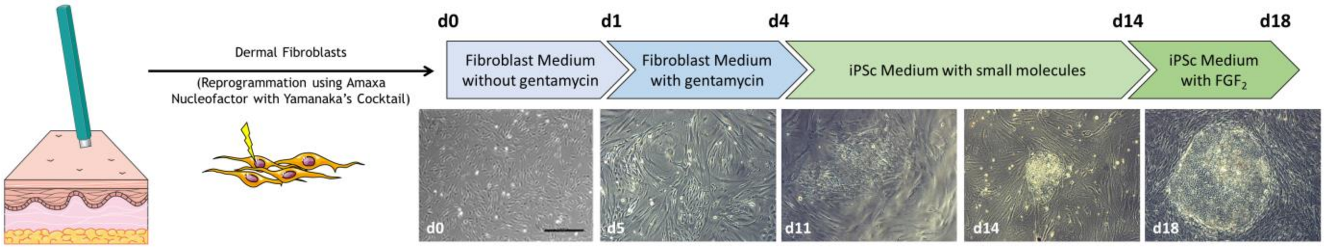

Figure S1. Schematic representation of hiPSC induction. Dermal fibroblasts were reprogrammed with Yamanaka's cocktail (Oct4, Sox2, Klf4, c-Myc) into induced pluripotent stem cells. HiPSC clones were generated in 18 days and then amplified to passage 15.

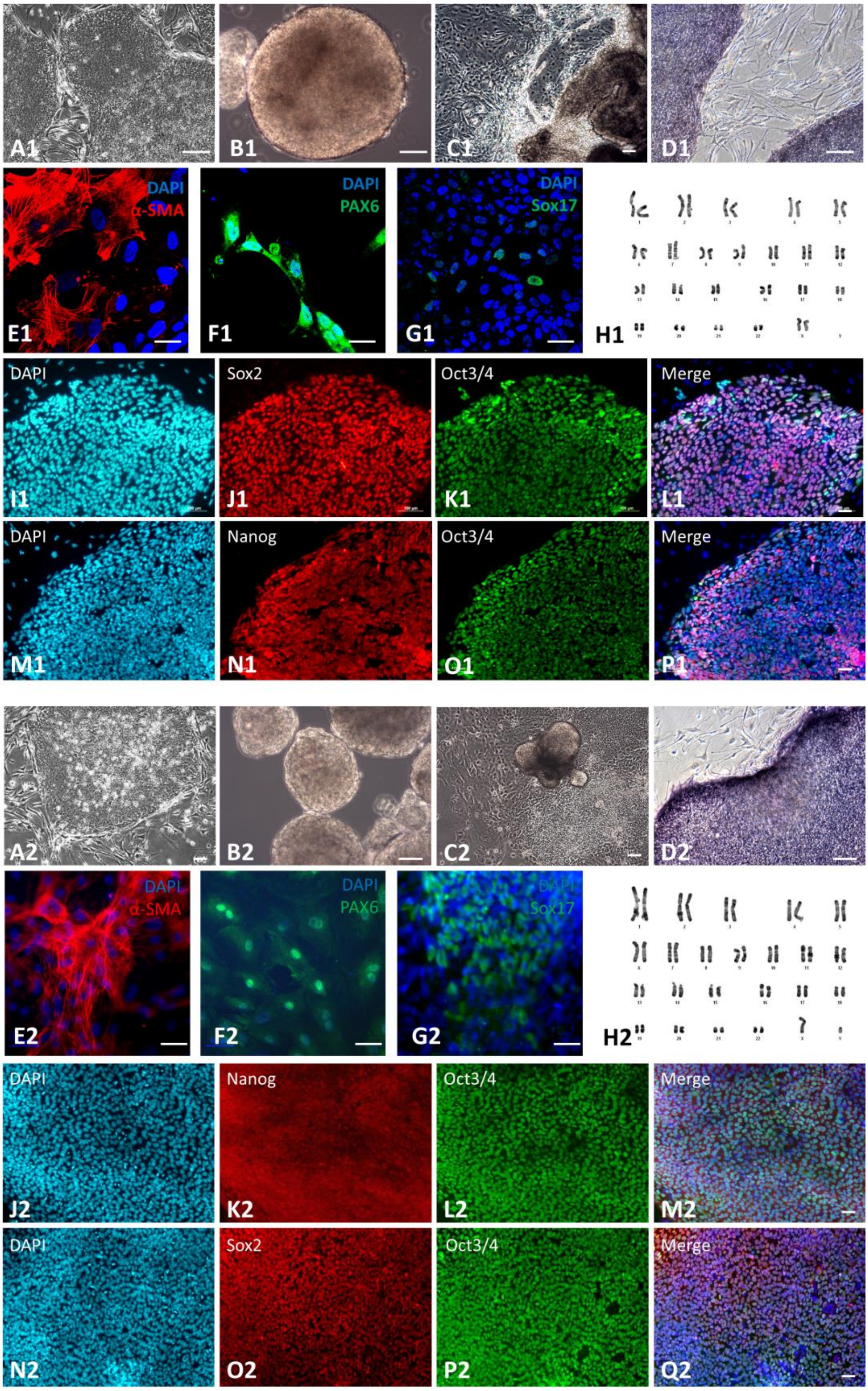

Figure S2. hiPSC generated from a healthy control (A1- P1) and a CMT2 patient (A2-P2). HiPSC colonies have a typical morphology (A1, A2), with a nucleus/cytoplasma ratio of 1:1. Embryoïd bodies (B1, B2) could be differentiated into cell types from the three embryonic germ layers following spontaneous differentiation (C1, C2) and labelling with α-SMA ((E1, E2) mesoderma), PAX6 ((F1, F2) ectoderma), and Sox17 ((G1,G2) endoderma). HiPSCs expressed pluripotency markers, including Nanog, Oct3/4, and Sox2 (I1-P1, I2, P2), were positive for alkaline phosphatase (D1, D2), and had normal karyotypes (H1, H2).

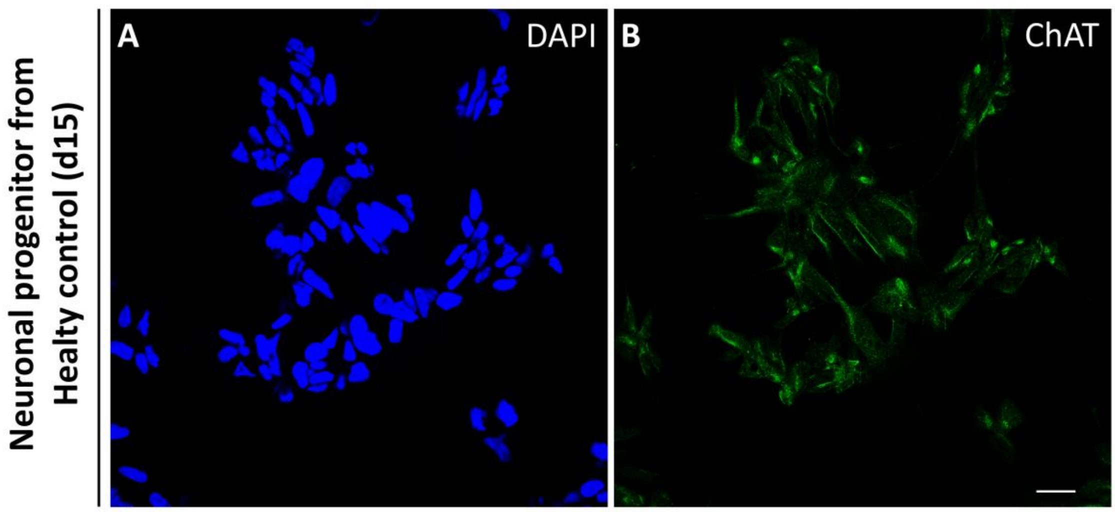

Figure S3. ChAT immunostaining performed at d15 on neuronal progenitors from a healthy control.

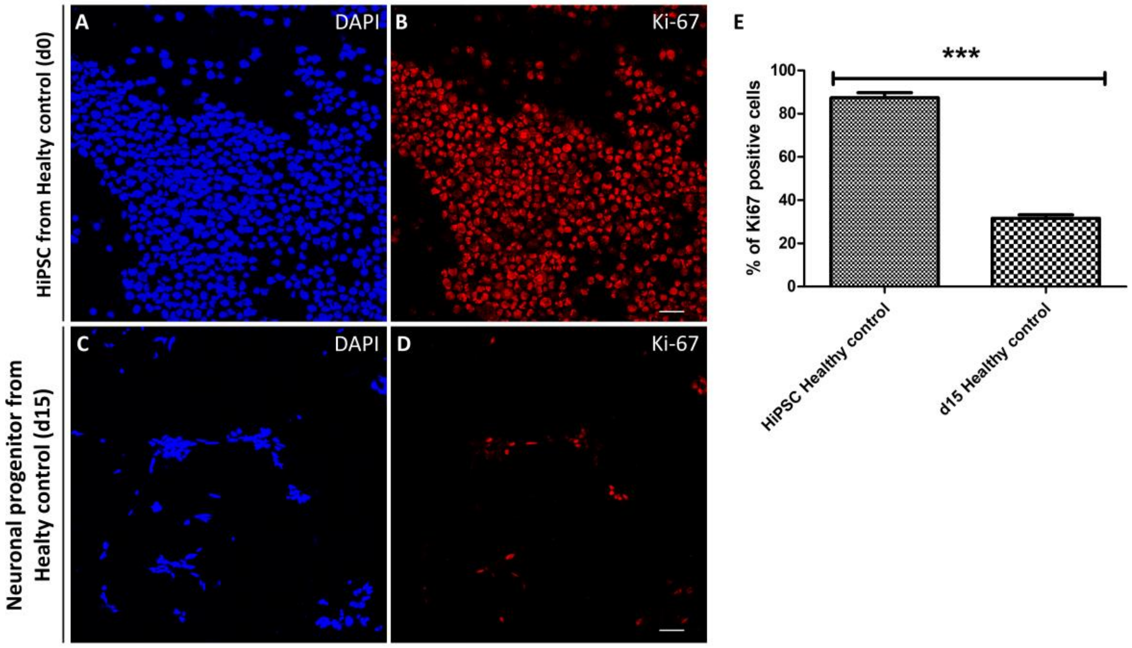

Figure S4. Ki-67 immunostaining performed at d0 (iPSC stage A-B, E) and d15 (neuronal progenitor C-D, E) from a healthy control. (E) Histograms showed the KI-67 positives cells betwenn hiPSCs (d0) and neuronal progenitor (d15) from a healthy control (Student T Test, n = 4 to 7, ***p<0.001).

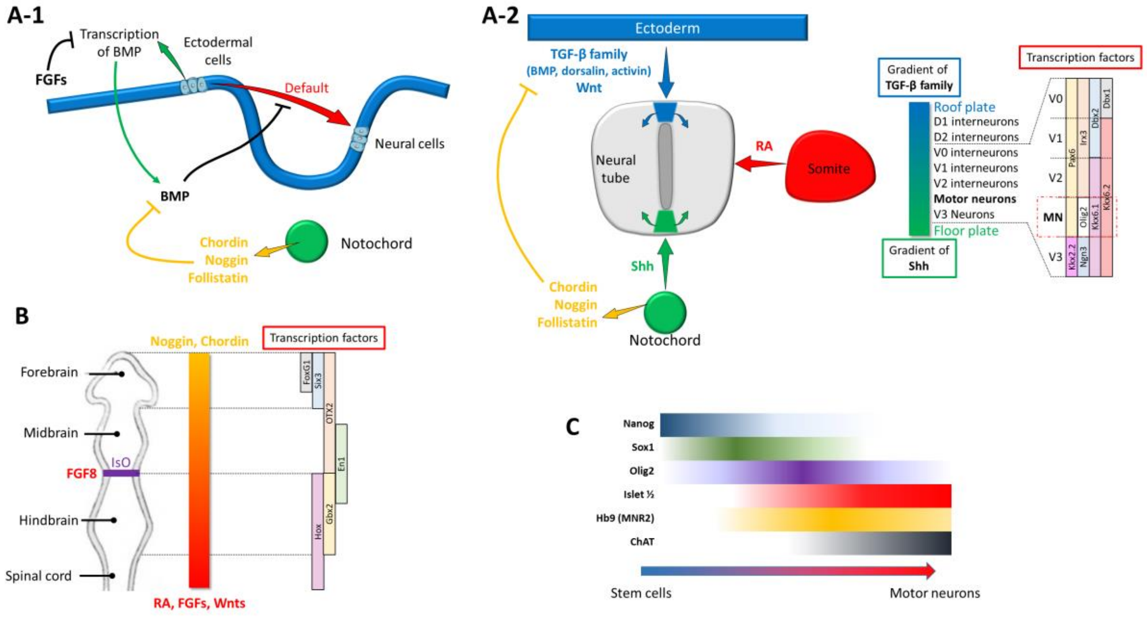

Figure S5. Factors involved in dorsal-ventral polarity during cord differentiation (A), factors involved in antero-posterior differentiation during neurulation (B), and MN markers during differentiation (C) adapted from Casarosa et al., 2013 and Davis-Dusenbery et al., 2014 [35,37]
